# Prediction of protein content in paddy rice (*Oryza sativa L.*) combining near-infrared spectroscopy and deep-learning algorithm

**DOI:** 10.3389/fpls.2024.1398762

**Published:** 2024-07-31

**Authors:** Ha-Eun Yang, Nam-Wook Kim, Hong-Gu Lee, Min-Jee Kim, Wan-Gyu Sang, Changju Yang, Changyeun Mo

**Affiliations:** ^1^ Department of Interdisciplinary Program in Smart Agriculture, Kangwon National University, Chuncheon, Republic of Korea; ^2^ Agriculture and Life Sciences Research Institute, Kangwon National University, Chuncheon, Republic of Korea; ^3^ Department of Crop Production and Physiology, National Institute of Crop Science, Rural Development Administration, Wanju, Republic of Korea; ^4^ Department of Agricultural Engineering, National Institute of Agricultural Science, Rural Development Administration, Wanju, Republic of Korea; ^5^ Department of Biosystems Engineering, Kangwon National University, College of Agriculture and Life Sciences, Chuncheon, Republic of Korea

**Keywords:** protein prediction, paddy rice, deep neural network (DNN), support vector regression (SVR), partial least square regression (PLSR), near-infrared spectroscopy (NIRS)

## Abstract

Rice is a staple crop in Asia, with more than 400 million tons consumed annually worldwide. The protein content of rice is a major determinant of its unique structural, physical, and nutritional properties. Chemical analysis, a traditional method for measuring rice’s protein content, demands considerable manpower, time, and costs, including preprocessing such as removing the rice husk. Therefore, of the technology is needed to rapidly and nondestructively measure the protein content of paddy rice during harvest and storage stages. In this study, the nondestructive technique for predicting the protein content of rice with husks (paddy rice) was developed using near-infrared spectroscopy and deep learning techniques. The protein content prediction model based on partial least square regression, support vector regression, and deep neural network (DNN) were developed using the near-infrared spectrum in the range of 950 to 2200 nm. 1800 spectra of the paddy rice and 1200 spectra from the brown rice were obtained, and these were used for model development and performance evaluation of the developed model. Various spectral preprocessing techniques was applied. The DNN model showed the best results among three types of rice protein content prediction models. The optimal DNN model for paddy rice was the model with first-order derivative preprocessing and the accuracy was a coefficient of determination for prediction, R_p_^2^ = 0.972 and root mean squared error for prediction, RMSEP = 0.048%. The optimal DNN model for brown rice was the model applied first-order derivative preprocessing with R_p_^2^ = 0.987 and RMSEP = 0.033%. These results demonstrate the commercial feasibility of using near-infrared spectroscopy for the non-destructive prediction of protein content in both husked rice seeds and paddy rice.

## 1 Introduction

Rice (*Oryza sativa L.*), with an annual production exceeding 400 million tons globally, is consumed as a staple food in Asia (Choi, 2007; Kim and Kim, 2023). In regions where rice constitutes the staple diet, a marked preference exists for rice of superior quality, characterized by specific content levels of certain rice components. The protein content in rice, a crucial component, defines its unique structural, physical, and nutritional attributes and plays a critical role in influencing rice’s water retention capacity, texture, taste, and ultimately its marketability (Jung, 2019). Consequently, protein content serves as a crucial quality indicator, affecting nutritional properties and quality assessment during harvesting and storage phases (Shi et al., 2022).

The Kjeldahl method is a common approach for determining grain protein content, including in rice (American Association of Cereal Chemists, 2000). However, it involves expensive equipment and skilled personnel, and is both time-consuming and cost-intensive. Moreover, traditional analytical methods frequently requisite pre-measurement processing steps such as drying, polishing, whitening, and milling.

To address these challenges, non-destructive spectroscopic analysis techniques are employed as a viable solution. These technologies facilitate the measurement of internal components without damaging the sample and are therefore extensively applied in food quality assessment (Wang and Paliwal, 2007). Near-infrared spectroscopy (NIRS), ultraviolet-visible (UV-Vis) spectroscopy, hyperspectral imaging, and Raman spectroscopy are among the techniques employed for quality measurement in agricultural products. NIRS, in particular, is favored for its environmental friendliness and capability to analyze multiple samples rapidly; therefore, it is utilized across various crop quality evaluation methods (Perez-Marin et al., 2019; Sharabiani et al., 2019; Teye et al., 2019; Mancini et al., 2020; Teye et al., 2020; Najjar and Abu-Khalaf, 2021; Khorramifar et al., 2022; Wang et al., 2022).

Near-infrared spectroscopy (NIRS) is a quantitative analysis method based on the principle that specific functional groups (such as O-H, N-H, and C-H) absorb near-infrared (NIR) light, causing vibrational overtones and combination vibrations. In the NIR region, absorption bands are caused by overtone and bond vibrations of the molecule, and follow the Beer-Lambert law, which states that the degree of absorption of light is proportional to the concentration of functional groups in the sample. The NIR region refers to the wavelength range of 800-2,500 nm (Beć et al., 2021). In the case of agricultural foods, the main components of fat (C-H), moisture (O-H), and protein (N-H, S-H) absorb near-infrared rays; thus, the components can be analyzed simultaneously using near-infrared spectroscopy (Williams and Norris, 1987). To date, several researchers have measured protein components in milled brown, white, whole grain brown, and white rice via NIRS (Delwiche et al., 1996; Shu et al., 1999; Kawamura et al., 2003; Kim et al., 2008; Bagchi et al., 2016; Fazeli Burestan et al., 2021). The application of NIRS for measurement exhibits promising potential to supplant conventional wet analysis methods in rice protein analysis. Analytical methodologies employing NIRS encompass multivariate analysis, machine learning, and deep learning. Although machine learning has conventionally served as a prevalent analytical tool for evaluating agricultural product quality, since 2010, deep learning, an advanced analytical approach, has gained traction in the realm of agricultural product quality assessment (Zhang et al., 2021).

Machine learning techniques such as partial least square regression (PLSR), support vector regression (SVR), partial least square discriminant analysis (PLS-DA), artificial neural networks (ANNs), and random forest (RF) and deep learning techniques such as convolutional neural networks (CNNs) are employed to establish quantitative relationships between NIRS spectra and rice protein content (Wang and Paliwal, 2007; Lin et al., 2019; Chadalavada et al., 2022; Sampaio and Brites, 2022). Several studies have identified partial least squares regression (PLSR) as the optimal model for protein content detection in rice (Lin et al., 2019). Moreover, a study demonstrated that the support vector regression (SVR) model exhibited superior accuracy compared with the PLSR model in predicting wheat protein content (Kamboj et al., 2022). Additionally, another study indicated that the artificial neural network (ANN) model, characterized by its non-linear nature, outperformed the linear PLSR model in predicting rice protein content (Kang et al., 2021).

Despite extensive research, most studies have focused on processed forms of rice, such as brown rice, white rice, or powder, largely due to the interference from rice husks in measuring the reflectance spectrum. This poses challenges in detecting the chemical components of rice enclosed by husks. Furthermore, research on developing models to non-destructively analyze the protein content of rice products using deep learning techniques is scarce.

This study aims to develop a technology for measuring the protein content of paddy rice by applying machine learning techniques such as PLSR and SVR, along with deep learning techniques such as DNN. The near-infrared spectral characteristics of paddy rice and brown rice according to the protein content were investigated, and the machine learning and deep learning models to predict protein content were developed and their performance was compared.

## 2 Materials and methods

### 2.1 Experimental samples

**Figure 1:**
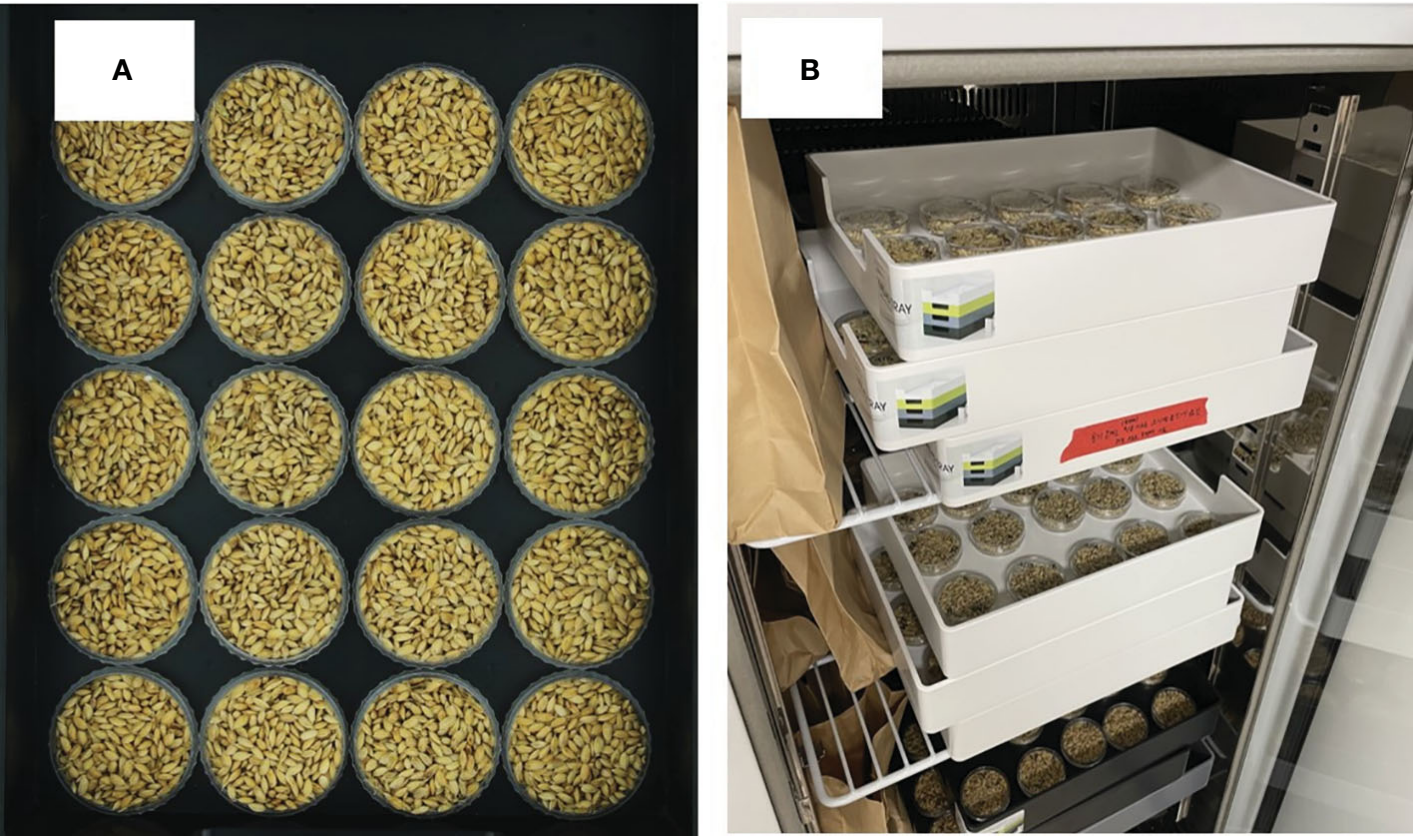
**(A)** Rice samples and **(B)** refrigerated storage.

The experimental samples comprised rice (*Oryza sativa* L. subsp. *Japonica*) of the Gyeonggi 13 variety, harvested in October 2022 from Gyeonggi-do province, Hwaseong City, South Korea (**Figure 1**). These samples were collected from 30 plots across four fields, yielding a total of 360 samples (4 fields × 30 plots per field × 3 samples per plot) following the threshing process. Each of the harvested samples was placed in petri dishes, 55 mm in diameter, with three samples generated for each plot, adding up to 360 paddy rice samples in total. In the case of brown rice samples, volume reduction occurs during the process removing the husk from the sample in the paddy rice. To secure the minimum sample amount for protein analysis, three samples produced per plot were combined into one sample. A total of 120 brown rice samples (4 fields × 30 plots per field) were produced. These samples were subsequently stored at a constant temperature of 20°C in a refrigerator.

### 2.2 NIR spectroscopy system and spectrum data acquisition

**Figure 2:**
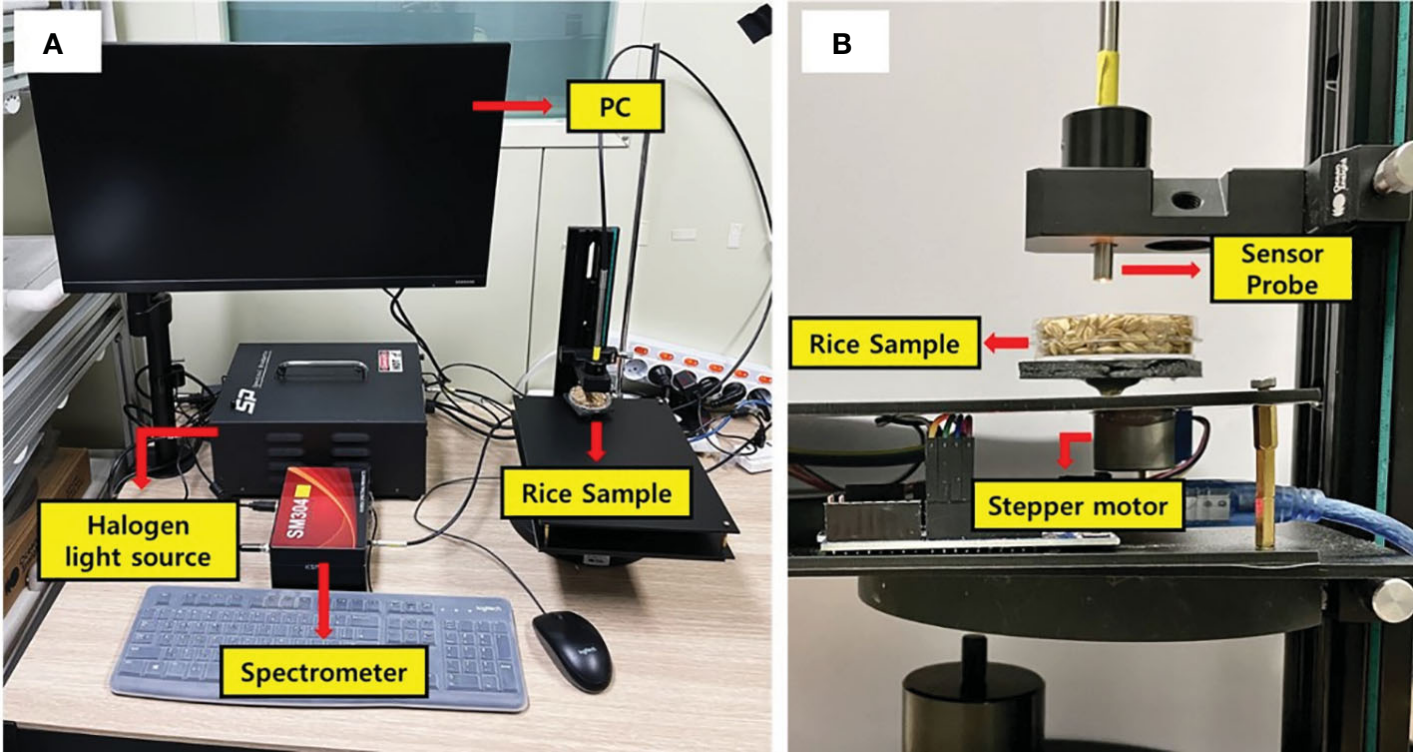
**(A)** Configuration of the NIR spectroscopic measurement system and **(B)** sample rotation unit driven by stepper motor.

For the acquisition of near-infrared (NIR) spectrum data, a near-infrared spectroscopy measurement system was utilized (**Figure 2**). The system comprised a near-infrared spectrometer (SM304, Korea Spectral Products, Seoul, South Korea), a 100 W tungsten-halogen lamp (ASBN-W100, KSP, South Korea) as the illumination source, and a stepper motor (28BYJ-48-5V, FSXSEMI, Shanghai, China) to facilitate the rotation of the sample holder. Rice samples underwent rotation in 36° increments via the stepper motor, enabling the measurement of reflectance spectra at 10 distinct spots on each sample. In this case, for paddy rice, spectra were collected at 5 points per sample from 360 samples, and for brown rice, 10 spectra were collected per sample from 120 samples. In order to further secure the spectrum of brown rice, where the number of samples was reduced to one third, twice as many locations as those of rice were measured. These measurements were conducted within the NIR range of 950–2200 nm, at a spectral resolution of 3.8 nm. The entire experiment was conducted in a dark room to eliminate potential spectral noise from external light sources.

To assess the spectral characteristics and prediction model accuracy with and without the rice husk, the NIR spectrum of paddy rice was initially measured. Subsequently, the rice husks were removed using a rice husk removal machine, and the absorbance of the de-husked (brown) rice was measured again under identical conditions.

The spectral reflectance data for each sample were normalized against a white reference and corrected using a dark reference. The dark reference measurements were taken without exposing the samples to the light source, whereas the white reference measurements utilized diffuse reflectance standards of 50% (Spectralon^®^ Diffuse Reflectance Standards, Labsphere, New Hampshire, USA). The formula used for reflectance calculation is present as Equation 1:


(1) $$R_{cal}=\frac{R_{s}–R_{dark}}{2.0\;R_{white}-{\;R}_{dark}}\;,$$

where R_white_ is the reflectance of white reference, R_dark_ is the reflectance of dark reference, and R_s_ is the reflectance of the sample.

### 2.3 Protein content analysis of rice

Following the acquisition of spectral data, the protein content in the rice samples was determined by converting the samples into a powder form and applying the micro Kjeldahl nitrogen quantification method (American Association of Cereal Chemists, 2000). This procedure involved heating the ground rice powder with sulfuric acid (H_2_SO_4_) and an oxidizing agent to facilitate digestion. The resultant ammonium sulfate was subsequently treated with an excess of alkali to generate ammonia, which was subsequently absorbed into a standard acid solution. The surplus acid was titrated with a standard alkali solution to determine the total nitrogen content. To perform the actual protein content analysis, three samples from the same harvest area were ground, combined into one sample, and analyzed in three replicates.

### 2.4 Development of rice protein content prediction models

For the development of rice protein content prediction models, models were established for both paddy rice and brown rice. The methodology involved the use of two machine learning models, PLSR and SVR, alongside a DNN model, a form of deep learning model. Subsequently, their performances were compared. The input for the PLSR, SVR, and DNN models comprised NIRS data, while the output was rice protein content data. The data used to develop these models included 1800 samples for paddy rice and 1200 samples for brown rice, with different zones sequentially selected from approximately two-thirds of the total measurement zone per sample. The averaged value was utilized for data analysis.

Spectral preprocessing technologies were employed to enhance performance and mitigate effects such as spectrum shape distortion, light scattering, and noise, which could arise from external environmental conditions (Bian, 2022). Various data preprocessing techniques, including Savitzky–Golay first-order and second-order derivatives, maximum normalization, mean normalization, range normalization, standard normal variate (SNV), and multiplicative scatter correction (MSC), were applied to compare model accuracy with and without data preprocessing. The efficacy of the protein content prediction model for rice products following each pretreatment method was compared and assessed. Reflectance spectrum preprocessing was executed using Unscrambler X (v10.4, CAMO SOFTWARE AS, Norway). To minimize data testing uncertainty and overfitting issues, 10-fold cross-validation was employed as the validation approach (de Oliveira Carneiro et al., 2023). The calibration dataset was divided into 10 distinct folds (subsets). In each iteration, one fold was used as the test set, while the remaining (10-1) folds were used as the training set to evaluate the model. This process was repeated 10 times, and the average performance was computed. The prediction of rice protein content was performed on independent prediction datasets using PLSR, SVR, and DNN models.

For the composition of the calibration dataset (training dataset) and validation dataset (test dataset), a stratified sampling distribution method was applied to randomly divide the data set and ensure statistical representativeness of the data. Using the stratified sampling distribution method, the distribution of protein content was similar in both the training and test sets. Through this, the protein distribution of rice in both data sets was preserved and bias was alleviated.

#### 2.4.1 Partial least squares regression model

The PLSR model, typically employed in chemometrics and spectral data analysis, identifies linear combinations of predictor (x, spectrum) and response variables (y, protein content) that exhibit a common structure (Geladi and Kowalski, 1986).

By maximizing the covariance between x and y, the PLSR algorithm alternates between regression and compression steps to derive a set of orthogonal factors termed optimal factors. These factors are assessed against a calibration set (Guo et al., 2019; Hao et al., 2019). Wavelengths were deemed significant when the b-coefficient surpassed thresholds set at the standard deviation of the values.

Equation 2 employed in the PLSR model is as follows. The PLSR model was developed using Unscrambler X software (v10.4, CAMO SOFTWARE AS, Norway). The dataset for developing the calibration model (calibration dataset) and the dataset for validating the developed calibration model (prediction dataset) were divided at the ratio of 7:3.

A calibration model was developed using the calibration dataset with 1260 samples for paddy rice and 840 samples for brown rice and 10-fold cross-validated. The model was subsequently verified using a prediction dataset, which consisted of unknown samples not utilized in the model’s development. The prediction datasets consisted of 540 samples for paddy rice and 360 samples for brown rice.


(2) $$\begin{array}{c} X=TP^{T}+E\\ Y=UQ^{T}+F\\ U=TB+H \end{array}$$

X(n×m) is the independent variable (spectral matrix); U(n×k) is the score matrix describing the dependent variable Y; T(n×k) is the score matrix describing the dependent variable X; P(m×k) is the eigenvalue matrix of the independent variable; Q(p×k) is the eigenvalue matrix of the dependent variable; E(n×m), F(n×p), and H(n×k) are the residual matrices; and B(k×k) is the regression coefficient of PLSR (Lee et al., 2022).

#### 2.4.2 Support vector regression model

SVR represents a sophisticated machine learning approach grounded in statistical learning theory. It operates within the domain of supervised learning, offering capabilities for pattern recognition and data analysis, with primary applications in classification and regression analysis. SVR, an adaptation of the SVM framework for regression, utilizes kernel functions to project input variables into a higher-dimensional feature space (Üstün et al., 2007). In this study, the linear regression (LR) kernel function was employed to develop a model for predicting rice protein content. Because linear models have faster learning and prediction speeds and are more economical than nonlinear models, the performance of two linear models (PLSR and SVR) in machine learning was compared. The SVR model was formulated using Unscrambler X (v10.4, CAMO SOFTWARE AS, Norway), with the calibration datasets and prediction datasets partitioned in a 7:3 ratio for model development as in 2.4.1.

#### 2.4.3 Deep neural network model

**Figure 3:**
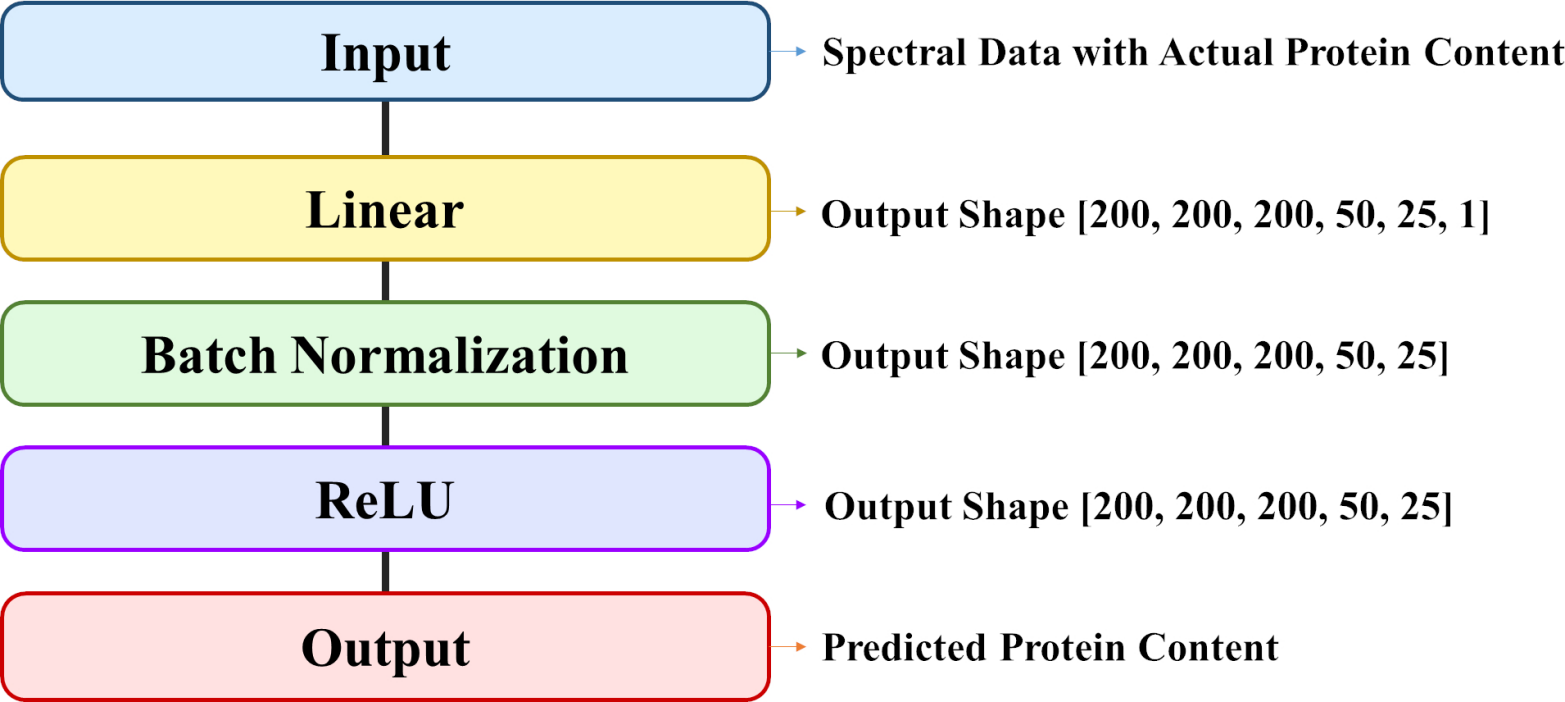
Architecture of DNN model.

The DNN model, a type of deep learning architecture, features a perceptron with multiple hidden layers, enabling the modeling of nonlinear relationships through specific activation functions associated with each layer. One of the main advantages of DNN is that in some cases, the step of feature extraction can be performed by the model itself. DNN models have significantly improved the state-of-the-art in many different sectors and industries, including agriculture (Liakos et al., 2018). This study utilized a neural network with five hidden layers, constructed using the Pytorch framework. The architecture of the DNN model includes an input layer (input), six linear layers (Linear1 to Linear6), five batch normalization layers (BatchNorm1d 1 to BatchNorm1d 5), five ReLu function layers (ReLu1 to ReLu5), and an output layer (Output) (**Table 1**) (**Figure 3**). The selected architecture consists of multiple fully connected layers with ReLU activation functions and batch normalization. This structure is designed to effectively capture and learn the complex relationships within the data. It enables the model to progressively learn from local features to more abstract, global features (Goodfellow et al., 2016). The input and output data correspond to the spectral data and protein content of the rice samples, respectively. Model development employed hyperparameters as outlined in **Table 2**. Adam, a widely adopted optimization algorithm in diverse deep learning models, was chosen for weight adjustments (Soydaner, 2020). The learning parameters—epoch, batch size, and learning rate—were determined to be 1000, 32, and 0.0005, respectively, through a trial-and-error approach. Model development leveraged Google Colab Pro and an NVIDIA Tesla T4 GPU, with the training datasets and prediction datasets partitioned in a 7:3 ratio for model development as in 2.4.1.

**Table 1:** Feedforward neural network with several hidden layers for regression tasks.

Layer (type)	Output Shape	Parameter
Linear-1	[-1, 200]	425,200
BatchNorm1d-1	[-1, 200]	400
ReLU-1	[-1, 200]	0
Linear-2	[-1, 200]	40,000
BatchNorm1d-2	[-1, 200]	400
ReLU-2	[-1, 200]	0
Linear-3	[-1, 200]	20,000
BatchNorm1d-3	[-1, 200]	200
ReLU-3	[-1, 200]	0
Linear-4	[-1, 50]	5,000
BatchNorm1d-4	[-1, 50]	100
ReLU-4	[-1, 50]	0
Linear-5	[-1, 25]	1,250
BatchNorm1d-5	[-1, 25]	50
ReLU-5	[-1, 25]	0
Linear-6	[-1, 1]	25

**Table 2:** Hyperparameters used in deep neural network (DNN).

Hyperparameter	Value
Learning Rate	0.001
Batch Size	32
Number of Epochs	5,000
Hidden Layer	5
Weight Decay	0.0000001
Loss Function	RMSE (Root Mean Square Error)
Optimizer	Adam

#### 2.4.4 Model performance evaluation

The evaluation of model performance involved comparing actual rice protein content against predictions derived from calibration and 10-fold cross-validation or independent prediction datasets across PLSR, SVR, and DNN models. The efficacy of the prediction model was gauged through various statistical measures, including the coefficient of determination for calibration (*R_c_* ^2^), cross-validation (*R_v_^2^*), and prediction (*R_p_* ^2^), alongside the root mean squared error for calibration (RMSEC), cross-validation (RMSEV), and prediction (RMSEP). The selection of the optimal model was based upon achieving the highest *R_p_* ^2^ and the lowest RMSEP. The formulas for each statistical measure, as represented in Equation 3, are provided for assessment.


(3) $$\begin{array}{c} R^{2}=1-\frac{\sum_{i=1}^{n}{\left(y_{i}-\hat{y_{i}}\right)}^{2}}{\sum_{i=1}^{n}{\left(y_{i}-\bar{y}\right)}^{2}},\\ RMSE=\sqrt{\frac{\sum_{i=1}^{n}{\left(y_{i}-{\hat{y}}_{i}\right)}^{2}}{n}},\\ bias=\sum_{i=1}^{n}\frac{{\hat{y}}_{i}-y_{i}}{n}, \end{array}$$

where $y_{i}$ and $\hat{y_{i}}$ are the reference and predicted values of the target variables in the sample, respectively; $\bar{y}$ is the mean of reference values, while n is the number of samples.

## 3 Results

### 3.1 Rice protein content analysis

The analysis of rice protein content in this study revealed that the protein concentration within the sampled rice, specifically the Gyeonggi No. 13 variety, was in the range 6.33–7.86 g/100 g, with an average content of 7.26 g/100 g (**Table 3**). This average is closely aligned with the national standard for brown rice protein content, which is 7.33 g/100 g as per the Korea National Standard Food Ingredients Table, indicating that the Gyeonggi No. 13 rice exhibits protein levels within the expected range for high-quality rice.

**Table 3:** Protein content of rice. (N=120).

	Min.	Max.	Avg.	Std.
Protein (g/100 g)	6.33	7.86	7.26	0.29

### 3.2 Near-infrared spectrum characteristics of paddy and brown rice

**Figure 4:**
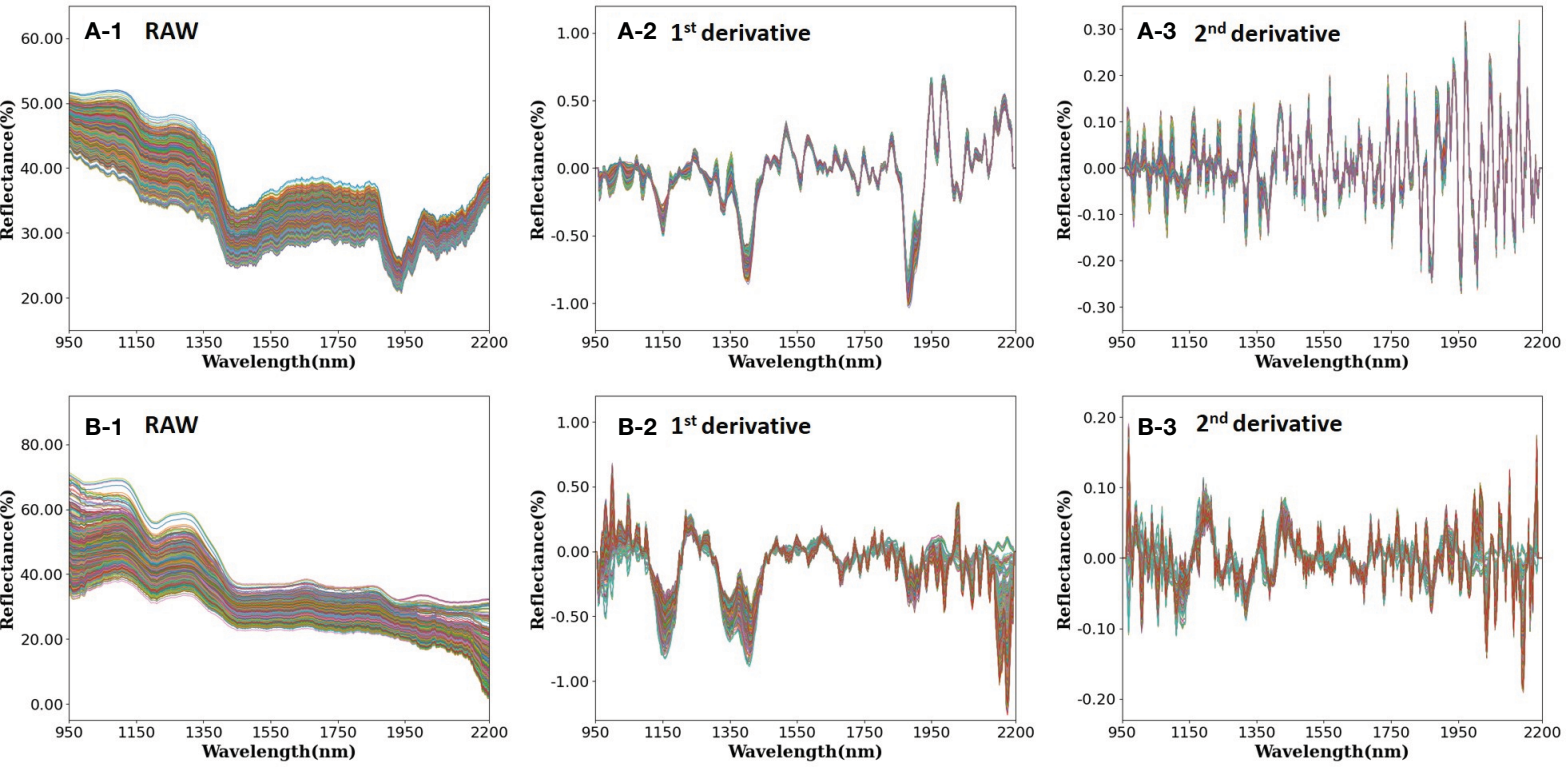
**(A)** Paddy rice and **(B)** brown rice reflectance with applied main preprocessing [(1) raw, (2) 1^st^ order derivative, and (3) 2^nd^ order derivative].

In the examination of the NIR reflectance spectra of rice samples, both with and without the rice husk (paddy and brown rice, respectively), notable differences in reflectance were observed based on the presence of the rice husk (**Figure 4**). The spectral analysis demonstrated similar trends for paddy rice and brown rice between 950 nm and 1400 nm, diverging significantly in the wavelength range of 1400–2200 nm. Specifically, brown rice exhibited lower reflectance in the wavelength range of 1400–2200 nm, a finding consistent with prior research (Weng et al., 2023).

The absorption peak observed at 985 nm within the NIR spectrum correlates with the N-H bond stretching vibration of protein (Wadood et al., 2019). Similarly, the absorption peak detected at 1200 nm is attributed to the C-H stretching vibration of lipids, starch, and proteins (Wadood et al., 2019; Wei et al., 2021). Moreover, absorption peaks noted at 1460 nm and 1940 nm are associated with the bending vibration of the O-H bond in water (Rohaeti and Rafi, 2017). Notably, the absorption peak of brown rice manifests as notably gentler than that of paddy rice within the wavelength bands of 1450 nm and 1950 nm. Thus, in this study, spectral analysis was conducted utilizing samples in the paddy rice state, with subsequent removal of the rice husk following complete desiccation to generate brown rice samples. Consequently, a reduction in moisture content was observed during this process.

The spectral trough observed at 1925 nm corresponds to the O-H single bond stretching vibration of cellulose and starch, alongside the C=O stretching vibration of protein secondary amide double bonds. Comparatively, the absorption peak evident in the spectrum of paddy rice surpasses that of brown rice, attributable to the presence of cellulose, a constituent of rice husk (Weng et al., 2023). Additionally, the absorption peak in the range 2000–2250 nm correlates with lignin, the principal constituent of plant xylem, thereby leading to a higher absorption rate in the spectrum of paddy rice with husk compared with husk-removed brown rice (Kästner et al., 2022).

**Figure 5:**
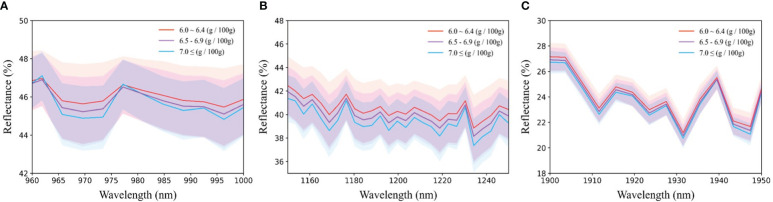
Average reflection spectrum around protein-related wavelength; **(A)** 985nm, **(B)** 1200nm, and **(C)** 1925nm.

**Figure 5** presents the average spectrum categorized by protein content, alongside the spectral range encompassing 985 nm, 1200 nm, and 1925 nm, all of which are linked to protein content. Notably, a negative correlation exists between protein content and spectral reflectance of the sample, indicating that higher protein content corresponds to heightened absorbance levels, considering the association of each wavelength band with protein content.

### 3.3 Development of protein content prediction model for paddy and brown rice

#### 3.3.1 PLSR model development for protein content prediction

A comprehensive analysis led to the development of 16 PLSR models aimed at predicting the protein content in both paddy and brown rice samples (**Table 4**). The performance of these models was evaluated based on their calibration and validation metrics, with and without the application of spectral preprocessing techniques.

**Table 4:** PLSR model performance results for the predicting the protein content of rice.

Rice Type	Preprocessing	$\;R_{c}^{\;2}$	RMSEC (*g*/100*g*)	$\;R_{v}^{\;2}$	RMSEV (*g*/100*g*)	$\;R_{p}^{\;2}$	RMSEP (*g*/100*g*)	Optimal factor
Paddy Rice	**Raw**	**0.928**	**0.395**	**0.927**	**0.396**	**0.918**	**0.082**	14
	Mean Normalization	0.939	0.073	0.921	0.083	0.918	0.082	14
	Range Normalization	0.937	0.074	0.918	0.085	0.919	0.081	14
	Maximum Normalization	0.934	0.076	0.916	0.086	0.914	0.084	14
	1^st^ order derivative	0.933	0.076	0.916	0.085	0.904	0.088	14
	2^nd^ order derivative	0.935	0.075	0.919	0.084	0.885	0.097	14
	**MSC**	**0.940**	**0.072**	**0.922**	**0.083**	**0.918**	**0.082**	14
	SNV	0.932	0.077	0.915	0.086	0.913	0.084	14
Brown Rice	**Raw**	**0.947**	**0.066**	**0.926**	**0.079**	**0.928**	**0.079**	14
	Mean Normalization	0.942	0.069	0.921	0.081	0.925	0.081	13
	Range Normalization	0.943	0.069	0.922	0.081	0.923	0.082	14
	Maximum Normalization	0.945	0.068	0.924	0.080	0.926	0.080	13
	1^st^ order derivative	0.944	0.069	0.921	0.081	0.910	0.089	14
	2^nd^ order derivative	0.942	0.070	0.917	0.083	0.917	0.085	13
	MSC	0.944	0.069	0.922	0.081	0.927	0.080	13
	**SNV**	**0.946**	**0.067**	**0.925**	**0.079**	**0.928**	**0.080**	14

The meaning of the bold values indicates the preprocessing methods that achieved the best prediction performance along with their corresponding results.

For paddy rice, the models that demonstrated the most robust predictive capabilities were those without preprocessing and those that underwent MSC preprocessing. The calibration values for the model without preprocessing and the MSC-preprocessed model were characterized by coefficients of determination (*R_c_* ^2^) of 0.928 and 0.940, and RMSEC of 0.395 and 0.072, respectively. However, the validation metrics revealed a slight advantage in applying MSC preprocessing, with coefficients of determination for validation (*R_v_* ^2^) of 0.927 and 0.922, and RMSEV of 0.396 and 0.083, respectively, indicating a lower RMSEV with preprocessing. The prediction performance for unknown samples, as measured by the coefficient of determination of prediction (*R_p_* ^2^) and RMSEP, was identical for both models at 0.918 and 0.082, respectively.

**Figure 6:**
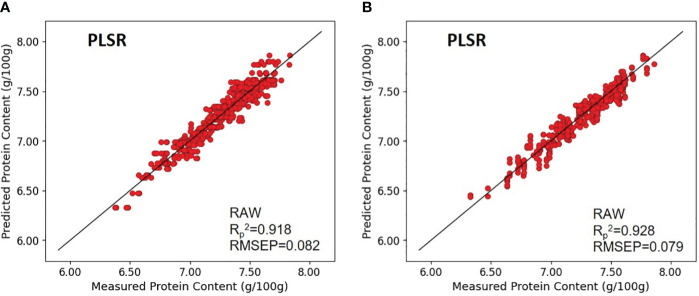
Validation results of the optimal PLSR model with prediction datasets: **(A)** paddy rice and **(B)** brown rice.

In the context of brown rice, models without preprocessing and those subjected to SNV preprocessing exhibited the highest predictive accuracy. The calibration values for these models exhibited (*R_c_* ^2^) of 0.947 and 0.946, and RMSEC of 0.066 and 0.067, respectively. The validation metrics for the model without preprocessing and the model with MSC preprocessing demonstrated comparable performance, with (*R_v_* ^2^) of 0.926 and 0.925, and RMSEV of 0.079 for both models. The prediction performance on unknown samples yielded (*R_p_* ^2^) and RMSEP of 0.928 and 0.079 for the unprocessed model, and 0.928 and 0.080 for the MSC-preprocessed model, respectively, indicating comparable predictive capabilities (**Figure 6**).

The analysis revealed that for both paddy and brown rice, the models without preprocessing performed comparably to those with the optimal preprocessing technique. Furthermore, while the brown rice protein content prediction models exhibited slightly higher overall performance than their paddy rice counterparts, the prediction accuracy between the two was nearly equivalent, highlighting the effectiveness of PLSR models in predicting rice protein content irrespective of rice type or preprocessing application.

**Figure 7:**
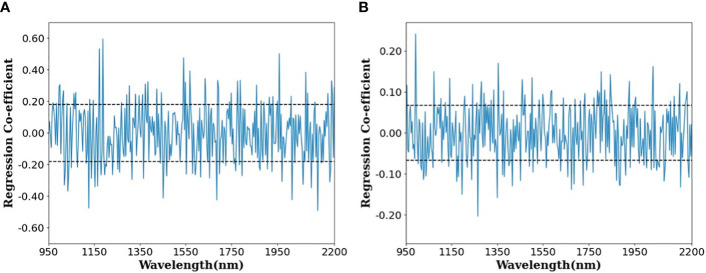
Regression coefficient plot of the optimal PLSR models for predicting protein content in **(A)** paddy rice and **(B)** brown rice.

**Figure 7** shows the regression coefficients that are instrumental in determining the effective wavelength within the optimal PLSR model—specifically, the raw data model devoid of any preprocessing—for the purpose of predicting the protein content in paddy and brown rice. An effective wavelength, deemed capable of delineating variations in protein parameters, is recognized as either a positive or negative regression coefficient when the standard deviation surpasses the predetermined threshold (illustrated by the dotted line). Previous research has identified a spectral band indicative of protein content in the vicinity of 1570, 985, 1200, and 1925 nm (Kim et al., 2008; Weng et al., 2023). The current study reveals significant positive peaks correlating with protein content around the wavelengths of approximately 985 and 1950 nm, whereas negative peaks were observed in the ranges of approximately 985–1000 nm and 1150–1200 nm. These findings align with those of a preceding study, which confirmed 985, 1200, and 1925 nm as principal wavelengths for protein content identification (Weng et al., 2023). Furthermore, the wavelength band near 1450 nm is posited to relate to O-H bonds (Rohaeti and Rafi, 2017).

#### 3.3.2 SVR model development for protein content prediction

A comprehensive study led to the development of 16 SVR models to forecast protein content in samples of paddy rice and brown rice, as detailed in **Table 5**. Within the SVR framework, the model utilizing first-order derivative preprocessing emerged as the most effective for predicting protein content in paddy rice.

**Table 5:** SVR model performance results for the predicting the Protein content of rice.

Rice Type	Preprocessing	$\;R_{c}^{\;2}$	RMSEC (*g*/100*g*)	$\;R_{v}^{\;2}$	RMSEV (*g*/100*g*)	$\;R_{p}^{\;2}$	RMSEP (*g*/100*g*)	Kernel type
Paddy Rice	Raw	0.659	0.172	0.749	0.152	0.622	0.176	linear
	Mean Normalization	0.930	0.078	0.894	0.097	0.903	0.070	linear
	Range Normalization	0.895	0.096	0.844	0.117	0.859	0.089	linear
	Maximum Normalization	0.583	0.200	0.485	0.214	0.576	0.164	linear
	**1^st^ order derivative**	**0.952**	**0.065**	**0.928**	**0.079**	**0.932**	**0.062**	**linear**
	2^nd^ order derivative	0.949	0.067	0.925	0.081	0.929	0.066	linear
	MSC	0.944	0.070	0.916	0.086	0.852	0.088	linear
	SNV	0.949	0.067	0.927	0.08	0.926	0.066	linear
Brown Rice	Raw	0.816	0.127	0.817	0.132	0.806	0.134	**linear**
	Mean Normalization	0.941	0.075	0.909	0.090	0.916	0.147	linear
	Range Normalization	0.796	0.138	0.762	0.148	0.779	0.203	linear
	Maximum Normalization	0.576	0.198	0.468	0.212	0.574	0.089	linear
	1^st^ order derivative	0.958	0.060	0.94	0.071	0.931	0.078	linear
	**2^nd^ order derivative**	**0.958**	**0.060**	**0.946**	**0.068**	**0.943**	**0.071**	**linear**
	MSC	0.964	0.056	0.941	0.071	0.945	0.070	linear
	SNV	0.962	0.057	0.942	0.07	0.947	0.068	linear

The meaning of the bold values indicates the preprocessing methods that achieved the best prediction performance along with their corresponding results.

**Figure 8:**
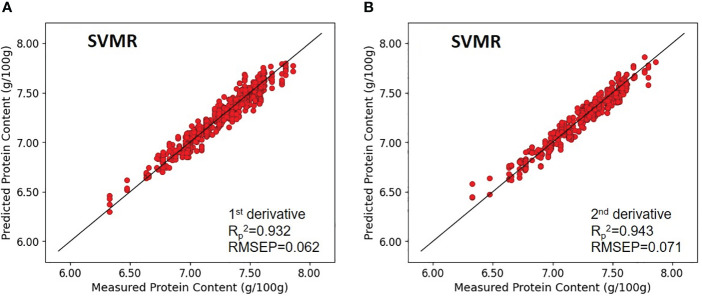
Validation results of the optimal SVMR model with prediction datasets: **(A)** paddy rice and **(B)** brown rice.

Specifically, the model’s coefficient of determination (*R_c_* ^2^) were 0.659 and 0.952, for paddy rice, without any pretreatment and with first-order derivative pretreatment, respectively, and the RMSEC were 0.172 and 0.065, without any pretreatment and with first-order derivative pretreatment, respectively. Regarding cross-validation results, the coefficient of determination for validation (*R_v_* ^2^) were 0.749 and 0.928, for paddy rice, without any pretreatment and with first-order derivative preprocessing and RMSEV were 0.152 and 0.079, without any pretreatment and with first-order derivative pretreatment, respectively. The prediction performance on unknown samples yielded (*R_p_* ^2^) and RMSEP values of 0.622, 0.176 for the unprocessed model, and 0.932, 0.062 for the first-order derivative -preprocessed model, respectively (**Figure 8**).

In brown rice, the protein-content prediction model employing second-order derivative preprocessing demonstrated superior performance. The calibration values for these models exhibited (*R_c_* ^2^) of 0.816 and 0.958, and RMSEC of 0.127 and 0.060, respectively. validation metrics for the model without preprocessing and the model with second-order derivative preprocessing demonstrated comparable performance, with (*R_v_* ^2^) of 0.817 and 0.946, and RMSEV of 0.132 and 0.068, respectively. Further, verification with an unknown sample indicated that (*R_p_* ^2^) were 0.806 and 0.943, and RMSEP were 0.134 and 0.071, respectively. The SVR models exhibited enhanced predictive accuracy over the PLSR models. Application of preprocessing techniques to both paddy and brown rice samples markedly improved model performance compared with their unprocessed counterparts. A comparison between the optimal model performances for paddy and brown rice, akin to the findings from the PLSR model development, indicated that the protein content prediction model for brown rice outperformed that for paddy rice. Without preprocessing, the (*R_p_* ^2^​) and RMSEP for the protein content prediction models for paddy rice and brown rice were 0.622 and 0.176 and 0.806 and 0.134, respectively, demonstrating superior performance of the brown rice model. Upon implementing optimal preprocessing, the (*R_p_* ^2^) and RMSEP for the protein content prediction models for paddy rice and brown rice improved to 0.932 and 0.062 and 0.943 and 0.071, respectively, thereby narrowing the accuracy gap observed without preprocessing (**Figure 8**).

#### 3.3.3 DNN model development for protein content prediction

Sixteen DNN models were devised to predict the protein content in samples of paddy rice and brown rice, as delineated in **Table 6**. For paddy rice, the model that incorporated first-order derivative preprocessing emerged as the most efficacious. The calibration values for the paddy rice model without preprocessing and the first-order derivative-preprocessed model were characterized by coefficients of determination (*R_c_* ^2^) of 0.934 and 0.978, and RMSEC of 0.071 and 0.040, respectively. Regarding validation results, the coefficient of determination for validation (*R_v_* ^2^) of 0.931 and 0.971, and RMSEV of 0.076 and 0.047, respectively. The prediction performance for unknown samples, as measured by the coefficient of determination of prediction (*R_p_* ^2^) of 0.936 and 0.972, and RMSEP of 0.074 and 0.048, respectively.

**Table 6:** DNN model performance results for the predicting the Protein content of rice.

Rice Type	Preprocessing	$\;R_{c}^{\;2}$	RMSEC (*g*/100*g*)	$\;R_{v}^{\;2}$	RMSEV (*g*/100*g*)	$\;R_{p}^{\;2}$	RMSEP (*g*/100*g*)
Paddy Rice	Raw	0.934	0.071	0.931	0.076	0.936	0.074
	Mean Normalization	0.980	0.039	0.965	0.053	0.967	0.053
	Range Normalization	0.891	0.093	0.843	0.111	0.843	0.113
	Maximum Normalization	0.969	0.049	0.966	0.053	0.966	0.054
	**1^st^ order derivative**	**0.978**	**0.040**	**0.971**	**0.047**	**0.972**	**0.048**
	2^nd^ order derivative	0.988	0.048	0.971	0.028	0.971	0.049
	MSC	0.984	0.036	0.96	0.055	0.963	0.056
	SNV	0.977	0.042	0.96	0.058	0.959	0.008
Brown Rice	Raw	0.931	0.073	0.917	0.083	0.916	0.082
	Mean Normalization	0.961	0.057	0.954	0.059	0.944	0.065
	Range Normalization	0.921	0.079	0.917	0.082	0.924	0.077
	Maximum Normalization	0.896	0.09	0.796	0.126	0.79	0.129
	**1^st^ order derivative**	**0.986**	**0.034**	**0.976**	**0.042**	**0.987**	**0.033**
	2^nd^ order derivative	0.98	0.038	0.98	0.04	0.982	0.038
	MSC	0.971	0.046	0.974	0.045	0.975	0.044
	SNV	0.973	0.046	0.977	0.043	0.979	0.041

The meaning of the bold values indicates the preprocessing methods that achieved the best prediction performance along with their corresponding results.

**Figure 9:**
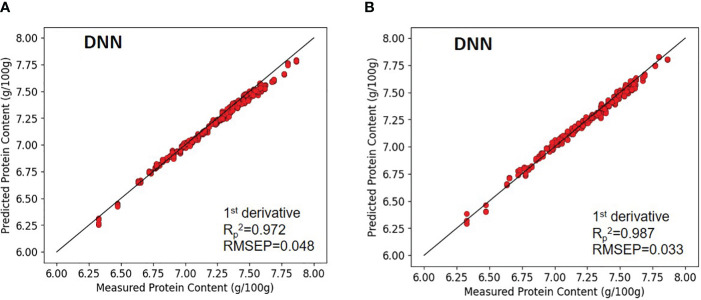
Validation results of the optimal DNN model with prediction datasets: **(A)** paddy rice and **(B)** brown rice.

In the case of brown rice, as paddy rice, the model applying first-order derivative preprocessing demonstrated superior performance. The calibration values for the paddy rice model without preprocessing and the first-order derivative-preprocessed model were characterized by coefficients of determination (*R_c_* ^2^) of 0.931 and 0.986, and RMSEC of 0.073 and 0.034, respectively. Regarding validation results, the coefficient of determination for validation (*R_v_* ^2^) of 0.917 and 0.976, and RMSEV of 0.083, 0.042, respectively. The prediction performance on unknown samples yielded (*R_p_* ^2^) and RMSEP of 0.916 and 0.082 for the without-preprocessed model, and 0.987 and 0.033 for the first-order derivative-preprocessed model, respectively (**Figure 9**).

The DNN models demonstrated higher predictive accuracy compared to PLSR and SVR. The optimal DNN models for paddy rice and brown rice achieved (*R_v_* ^2^) and (*R_p_* ^2^) values between 0.971 and below 0.987, with RMSEC and RMSEP values ranging from above 0.033 to below 0.048. Preprocessing significantly enhanced performance for both paddy rice and brown rice samples compared to the non-preprocessed models. In the comparative analysis of optimal model performance, the model performance for brown rice was higher but both types of rice exhibited relatively strong predictive capabilities for protein content.

#### 3.3.4 Performance evaluation of optimal model for predicting protein content of rice

The optimal models for predicting protein content in paddy rice and brown rice were evaluated using a prediction dataset not involved in the model development process, as illustrated in **Figures 7**–**9**. Among the evaluated models, the DNN model demonstrated superior performance compared with the PLSR and SVR models. Specifically, the coefficients of prediction (*R_p_* ^2^) for the optimal PLSR (RAW), SVR (first-order derivative), and DNN (first-order derivative) models for paddy rice were 0.918, 0.932, and 0.972, respectively. For brown rice, the corresponding R_p_^2^ values for the optimal PLSR (RAW), SVR (second-order derivative), and DNN (first-order derivative) models were 0.928, 0.943, and 0.987, respectively. The performance of the SVR model surpassed that of the PLSR model, while the DNN model exhibited the most substantial performance enhancement. In the case of paddy rice, the R_p_^2^ values for the optimal PLSR, SVR, and DNN models varied from 0.918 to 0.972, with RMSEP values ranging from 0.048 to 0.082 g/100g. For brown rice, the R_p_^2^ values for the optimal PLSR, SVR, and DNN models was in the range 0.928–0.987, with RMSEP values ranging from 0.033 to 0.079 g/100g. Although the prediction accuracy for the protein content in paddy rice was lower than that for brown rice, the models for both types of rice demonstrated nearly equivalent performance.

## 4 Discussion

This study demonstrates the feasibility of predicting the protein content in paddy rice with accuracy comparable to that of brown rice using NIR spectroscopy, alongside machine learning and deep learning techniques. The developed PLSR, SVR, and DNN models are capable of estimating the protein content in rice, with values ranging from 6.33 g/100g to 7.86 g/100g.

The application of spectral preprocessing techniques during model development significantly enhanced the prediction performance of the models. Preprocessing methods could effectively mitigate the impact of high-frequency noise, including the light scattering effect attributable to particles of various sizes and shapes. The implementation of Savitzky–Golay first and second order derivatives preprocessing on the SVR and DNN models notably improved prediction accuracy, as depicted in **Figures 8**, **9**. These findings are in alignment with prior research indicating the superior efficacy of Savitzky–Golay preprocessing over other methods in most machine learning applications (Vestergaard et al., 2021). Upon comparing the prediction performance of models with and without rice husk, the PLSR, SVR, and DNN models were observed to demonstrate superior performance in predicting protein content in brown rice. These outcomes are attributed to interference from rice husk, corroborating findings from prior studies suggesting that rice husk exerts a specific influence on spectral photosensitivity, thereby acting as a disruptive factor in prediction (Li et al., 2015). Furthermore, the samples utilized in this study were directly harvested from rice fields and threshed using a rudimentary thresher, potentially resulting in coarser threshing compared to mechanized harvesting methods. Augmenting experiments to enhance mutual correlation could enhance prediction accuracy in future endeavors.

The development outcomes of the PLSR and SVR machine learning models, along with the DNN deep learning model (**Figures 7**–**9**), showed comparable or superior accuracy compared to previous studies. For instance, Ma et al. reported R_p_^2^ and RMSEP values of 0.843, 0.44, and 0.829, 0.23, respectively, when predicting paddy rice protein content using PLSR and SVR models in hyperspectral imaging technology (Ma et al., 2021). Similarly, Lian et al. predicted white rice protein content utilizing PLSR and ANN models in NIRS, yielding R_p_^2^ and SEP values of 0.934, 0.157, and 0.824, 0.257, respectively (Lin et al., 2019). In this study, the DNN model demonstrated superior performance compared with the PLSR and SVR models. The inherent characteristics of the DNN model, comprising multiple hidden layers, facilitated the derivation of complex functional relationships between paddy rice and protein content more effectively than the machine learning models, PLSR and SVR. While PLS and SVM models exhibit linearity, DNN can learn complex patterns, including nonlinear relationships. This advantage likely enabled it to better capture the correlation between protein content and spectral data. Furthermore, while PLSR and SVR models in this research learn linear relationships, DNNs are capable of learning complex patterns, including nonlinear relationships. This distinction likely facilitated a more accurate capture of the correlation between protein content and spectral data.

While most extant studies have collected and analyzed spectral data employing husk-removed white or brown rice samples in powdered form (Kim et al., 2008; Xie et al., 2014; Bagchi et al., 2016; Fazeli Burestan et al., 2021; Mishra et al., 2021; Liu et al., 2023; Shi et al., 2023), this study utilized near-infrared spectrum obtained from paddy rice containing rice husk and husk-removed brown rice. The achievement of high prediction accuracy by employing deep learning analysis techniques highlights the significance of this approach. These findings show that the application of deep learning technology is an effective method in a wide range of agricultural product quality evaluation fields, especially non-destructive testing of paddy rice. Moreover, the findings indicate the potential for rapid and non-destructive prediction of paddy rice protein content using near-infrared spectroscopy, even in conditions involving freshly harvested rice with high moisture content and rice husk.

Furthermore, the technology holds promise for diverse applications. The development of a portable device could enable real-time assessment of rice quality on farms. Conversely, if developed as an indoor measuring device, it could facilitate quality determination during rice purchase and sale at rice processing centers (RPCs). Moreover, it could be utilized for immediate testing and research on rice quality post-harvest. By integrating the insights from this study into a combine-mounted system, real-time acquisition of rice data for quality assessment becomes feasible. Such data could inform nitrogen fertilizer application adjustments for each rice field area in subsequent years. These results can also be applied in the processing industry when selecting rice for specific protein content. Recent research has explored the use of Near-Infrared (NIR) spectroscopy and machine learning to measure protein content in a variety of samples, including not only rice but also edible insects and plant-based meat substitutes (Li et al., 2023; Xiao et al., 2023). This approach will holds promise as a nutritional analysis technology for addressing a range of food security issues.

Nonetheless, this study has limitations, particularly regarding the necessity for a wider range of predictable protein content to effectively apply this model in industrial settings. In order to apply these models to various rice varieties, acquiring spectral data and developing models for various rice varieties will be necessary. Additionally, the construction of big data will be necessary to strengthen model robustness to the variability of rice quality. As a future research direction, additional deep learning models other than DNN can be incorporated to compare prediction accuracy between models.

## 5 Conclusion

In this study, models for predicting the protein content in unhusked (paddy) rice and brown rice were developed employing PLSR, SVR, and DNN algorithms, in conjunction with NIR spectroscopy technology. The models were refined through the application of various spectral preprocessing techniques, including normalization, first-order derivative, second-order derivative, MSC, and SNV transformation, which were subsequently correlated with actual protein content values.

The prediction model for rice protein content (with overall protein contents ranging from 6.33–7.86 g/100 g) showed the high prediction performance in deep learning model (DNN) than machine learning model (PLSR, SVR). The optimal DNN model, with Savitzky–Golay first-order derivative preprocessing applied to paddy rice, attained Rp² of 0.972 and RMSEP of 0.048. Similarly, for brown rice, upon applying Savitzky–Golay first-order derivative preprocessing, the optimal DNN model achieved an Rp² of 0.987 and an RMSEP of 0.033.

The findings of this study highlight the potential for non-destructive measurement of protein content in paddy rice through the integration of machine learning and deep learning algorithms with NIR spectroscopy technology.

## Data availability statement

The original contributions presented in the study are included in the paper/supplementary material, and any further inquiries can be directed to the corresponding author.

## Author contributions

HY: Conceptualization, Methodology, Writing – original draft, Data curation, Formal analysis. NK: Conceptualization, Data curation, Investigation, Writing – original draft. HL: Data curation, Software, Writing – original draft. MK: Data curation, Investigation, Writing – original draft. WS: Resources, Writing – original draft. CY: Resources, Writing – original draft. CM: Conceptualization, Methodology, Project administration, Supervision, Writing – review & editing.
